# Comparative cytogenetics among populations of *Astyanax altiparanae* (Characiformes, Characidae, *Incertae sedis*)

**DOI:** 10.1590/S1415-47572009005000078

**Published:** 2009-12-01

**Authors:** Maressa Ferreira, Marcelo Ricardo Vicari, Edemar Furquim de Camargo, Roberto Ferreira Artoni, Orlando Moreira-Filho

**Affiliations:** 1 Laboratório de Citogenética, Departamento de Genética e Evolução, Universidade Federal de São Carlos, São Carlos, SPBrazil; 2 Departamento de Biologia Estrutural, Molecular e Genética, Universidade Estadual de Ponta Grossa, Ponta Grossa, PRBrazil

**Keywords:** cytotaxonomy, karyotype diversification, fish cytogenetics

## Abstract

Cytogenetic data are presented for *Astyanax altiparanae* populations from three Brazilian hydrographic systems. The chromosomal data obtained in *A. altiparanae* support the hypothesis of diploid number conservation. However, small differences in the karyotype formula and number of nucleolar organizer regions were observed in these populations. The apparent karyotypical similarity among the studied populations strongly suggests a close relationship among them with some chromosomal divergences due to gene flow restriction.

*Astyanax* is a genus from the family Characidae widely distributed throughout South and Central America and previously assigned to the subfamily Tetragonopterinae (Géry, 1977). This group has been recently considered *Incertae sedis* for not exhibiting consistent evidence of monophyletism (Lima *et al.*, 2003). *Astyanax altiparanae* is a typical species of the upper Paraná River Basin above Sete Quedas, Brazil (Garutti and Britski, 2000), with the exception of one population from a location in the Iguaçu River situated below this region (Graça and Pavanelli, 2002).

The cytogenetics of Neotropical fishes has greatly contributed to their systematics and taxonomy. There are numerous examples among *Astyanax*, mainly from the “*scabripinnis*” and “*fasciatus*” groups, in which the intraspecific cytogenetic variation among populations was shown to be much larger than that detected through morphological systematics, thereby forming true species complexes (Moreira-Filho and Bertollo, 1991; Artoni *et al.*, 2006; Vicari *et al.*, 2008a).

The aim of this work was to characterize the karyotypes of three *A. altiparanae* populations and to compare them with those described in the literature. This comparative analysis could reveal population markers and help to establish cytosystematic, evolutionary and/or biogeographical relationships among the populations.

Forty-nine specimens of *A. altiparanae* were analyzed: nine specimens (five males and four females) from the Pântano Stream in the Tietê River Basin of the state of São Paulo; eight specimens (five males and three females) from the Feijão Stream in the Mogi-Guaçu River Basin in the state of São Paulo; and 32 specimens collected at the Salto Segredo hydroelectric power plant on the Jordão River in the Iguaçu River Basin in the state of Paraná. The procedures were approved by the Ethics Committee on Animal Experimentation of the Universidade Estadual de Ponta Grossa (Process no. 04741/08).

Chromosome preparations were obtained from anterior kidney cells using the *in vivo* colchicine treatment (Bertollo *et al.*, 1978). Nucleolar organizer regions (NORs) were detected after silver nitrate staining (Ag-NOR) according to Howell and Black (1980) and C-banding was obtained as described in Sumner (1972).

Fluorescent *in situ* hybridization (FISH) was performed with a biotinyled 18S rDNA probe from the fish *Prochilodus argenteus* (Hatanaka and Galetti Jr, 2004) and with a biotinyled 5S rDNA probe from the fish *Leporinus elongatus* (Martins and Galetti Jr, 1999). An Olympus BX50 epifluorescence microscope was used for the analysis.

Nearly 30 metaphases were analyzed per specimen to determine the diploid number, karyotype composition and Ag-NOR distribution. Chromosomes were classified as metacentric (m), submetacentric (sm), subtelocentric (st) and acrocentric (a) following Levan *et al.* (1964).

Fishes of the genus *Astyanax* have shown considerable karyotype diversity. The diploid and fundamental numbers (2n and FN) allied to some other chromosome markers, allowed cytotaxonomic and evolutionary inferences regarding these fishes. The *A. altiparanae* populations studied (Pântano Stream, Feijão Stream and Jordão River) have a diploid number of 2n = 50 chromosomes (Figure 1). However, differences in the fundamental numbers were found among the three populations analyzed. The specimens from the Pântano Stream displayed a FN = 88, with 6m+28sm+4st+12a. The Feijão Stream population had a FN = 94, with 6m+30sm+8st+6a, whereas the specimens from the Jordão River showed a FN = 92, with 6m+28sm+8st+8a (Figure 1, Table 1). In the Jordão River population, chromosome pair 2 was smaller than the same pair in the Pântano and Feijão Streams populations (Figure 1). Variations in diploid number were observed between populations of species from the *A. scabripinnis* and *A. fasciatus* complexes and resulted from chromosome translocations (Mantovani *et al.*, 2000; Artoni *et al.*, 2006; Vicari *et al.*, 2008a). Nevertheless, *A. altiparanae* exhibited a conserved 2n = 50 chromosomes in all the populations karyotyped (Table 1). Despite presenting the same 2n, different fundamental numbers were detected due to chromosome morphology variations. Non-Robertsonian rearrangements, such as pericentric inversions were suggested to explain the karyotype differences among *A. altiparanae* populations (Domingues *et al.* 2007).

**Figure 1:**
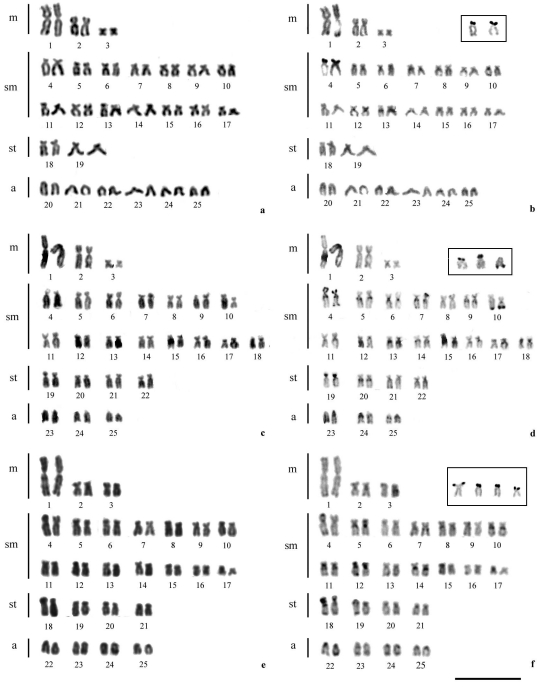
Karyotypes of *Astyanax altiparanae:* population from the Pântano Stream after conventional Giemsa staining (a) and sequential C-banding (b); in the inset, the chromosomes with Ag-NORs. Population from the Feijão Stream after conventional Giemsa staining (c) and sequential C-banding (d); in the inset, the chromosomes with Ag-NORs. Population from the Jordão River after conventional Giemsa staining (e) and sequential C-banding (f); in the inset, the chromosomes with Ag-NORs. The bar represents 10 μm.

After C-banding, heterochromatic blocks were observed in the centromeric or pericentromeric regions of all chromosome pairs and in the telomeric regions of some chromosomes with small differences among the analyzed populations (Figure 1b, d and f). This results are similar to findings previously described for other *A. altiparanae* populations (Fernandes and Martins-Santos, 2004; Domingues *et al.*, 2007). The small variations in constitutive heterochromatin distribution combined with the large variation in FN found among the *A. altiparanae* populations (Table 1) supports the hypothesis that this structural variation is not due to the amplification of heterochromatic sequences. Thus, the different FNs observed in *A. altiparanae* populations, sometimes even within the same hydrographic basin, such as the Tibagi, Paranapanema and Mogi-Guaçu Rivers, must be mainly due to pericentric inversions.

Silver-nitrate staining (Ag-NORs) and FISH with the 18S rDNA probe evidenced a single NOR-bearing pair in the population of the Pântano Stream, while the populations of the Feijão Stream and the Jordão River exhibited multiple terminal signals on different chromosomes (Figure 1, in the insets; and Figure 2a, c and e). Chromosome rearrangements and transference of ribosomal sites could cause these variations, but transposition events have been suggested as the main mechanism to explain the majority of cases of NORs variability in the genome of these animals (Mantovani *et al.*, 2000; Vicari *et al.*, 2008b). Together with variations in the fundamental number, Ag-NORs are important cytogenetic markers to determine differences among populations in this species (Pacheco *et al.*, 2001; Fernandes and Martins-Santos, 2004; 2006). Domingues *et al.* (2007) carried out a comparative cytogenetic and morphometric study on *A. altiparanae* populations from the upper Tibagi and Iguaçu Rivers. These authors found a similar karyotype macrostructure in the populations with differences in the number of major rDNA sites between populations. The Ag-NORs and 18S rDNA FISH data (Table 1) also revealed variations between populations of this species both within the same and from different hydrographic basins.

While variations in the number of 18S rDNA sites occurred between populations of *A.altiparanae*, only one signal in the proximal long arm of a sm chromosome was observed after FISH with the 5S rDNA probe (Figure 2b, d and e) (Fernandes and Martins-Santos, 2004; 2006; Domingues *et al.*, 2007; Peres *et al.*, 2008; present study). The 5S rDNA sites seemed to be conserved in the proximal region of the long arm of two chromosome pairs (one m and one a) in populations of the *A. scabripinnis* complex (Ferro *et al.*, 2001; Almeida-Toledo *et al.*, 2002; Mantovani *et al.*, 2005; Vicari *et al.*, 2008a). In *Astyanax* sp. D and in *A. janeiroensis,* the 5S rDNA site was located in the proximal region of the long arm of a single acrocentric pair, similar to what was found in the *A. scabripinnis* complex (Kantek *et al.*, 2007; Vicari *et al.*, 2008b, respectively). Mantovani *et al.* (2000) proposed that 5S rDNA sites in the proximal position of the long arm indicated a conserved pattern for these genes in *Astyanax.* Thus, the chromosomes with 5S rDNA sites would be the same among the *A. altiparanae* populations studied, but would diverge in morphology and number of 5S rDNA sites in other populations of the genus.

The cytogenetic data from the three *A. altiparanae* populations studied herein are similar to those from other populations of this species reported in the literature. This observation strengthens the hypothesis of a conserved 2n = 50. Nevertheless, differences in the karyotypic formula and in the number of nucleolar organizer regions were noticed when comparing the data from these populations to those already described for other populations. The apparent karyotype similarity strongly suggests a close relationship among the studied populations, but the small karyotypic differences detected indicate some evolutionary divergence due to gene flow restrictions.

## Figures and Tables

**Table 1:** Chromosome numbers (2*n*) in Argentinean species of Orchidaceae.

Taxon	2n	Locality and vouchers	2*n* - Previous references
*Aspidogyne kuczyynskii* (Porsh) Garay ^2^		Ctes. Ituzaingó, Santa Tecla, Almada 151	-
*Brassavola tuberculata* Hook	40	Mnes. Capital, Posadas, Hojsgaard 228	40 - Afzelius (1943), Blumenschein (1960)
*Campylocentrum neglectum* (Rchb. f. & Warm.) Cogn.	38	Chaco, San Fernando, Colonia Benítez, Insaurralde 676	38 - Dematteis and Daviña (1999)
*Catasetum fimbriatum* (C. Morren.) Lindl. & Paxton	108	Mnes. Capital, Posadas, Insaurralde 707	108 - Jones and Darker (1968), Dematteis and Daviña (1999)
*Corymborkis flava* (Sw.) Kuntze ^1^	56	Mnes. San Pedro, P. P. Saltos del Moconá, Daviña 208	-
*Cyclopogon callophyllus* (Barb. Rodr.) Barb. Rodr.^1^	28	Mnes. Capital, Nemesio Parma, Cerutti 74	-
	28	Ctes. Ituzaingó, Garapé, Cerutti 28	-
*C. congestus* (Vell.) Hoehne	32	Mnes. Candelaria, Aº Yabebiry, Almada 150	28, 32, 36 - Martínez (1981), Tanaka and Maekawa (1983)
	32	Mnes. Aristóbulo del Valle, Cuña Pirú, Hojsgaard 192	
*C. elatus* (Sw.) Schltr.	28	Mnes. Apóstoles, Aº Chimiray, Hojsgaard 341	28, 30, 45 - Martínez (1981), Felix and Guerra (2005)
	28	Ctes. Capital, Insaurralde 708	-
*C. oliganthus* (Hoehne) Hoehne & Schltr.^1^	64	Mnes. Apóstoles, Aº Chimiray, Hojsgaard 339	-
	64	Mnes. Capital, Posadas, Aº Zaimán, Cerutti 71	
*Cyrtopodium hatschbachii* Pabst ^1^	46	Mnes. Capital, Villa Lanús, Aº Zaimán, Almada 153	-
*C. palmifrons* Rchb. f. et Warm.^1^	46	Mnes. Capital, Posadas, Hojsgaard 354	-
*Eltroplectris schlechteriana* (Porto & Brade) Pabst	26	Mnes. Montecarlo, Honfi 1357	26 - Martínez (1985), Dematteis and Daviña (1999)
*Eurystyles actinosophila* (Barb. Rodr.) Schltr.^2^	56	Mnes. Capital, Honfi 1358	-
*Galeandra beyrichii* Rchb. f.^1^	54	Mnes. Capital, Garupá, Insaurralde 705	-
*Gomesa planifolia* (Lindl.) Klotzsch ex Rchb.f.^1^	56	Mnes. San Pedro, Piñalito, Guillén 492	-
*Habenaria bractescens* Lindl.^1^	44	Mnes. Capital, Garupá, Insaurralde 697	-
*Leptotes unicolor* Bar. Rodr.	40	Mnes. San Ignacio, Teyú Cuaré, Seijo 706	40 - Blumenschein (1960)
*Mesadenella cuspidata* (Lindl.) Garay	46	Mnes. Capital, Garupá, Hojsgaard 349	46 - Martínez (1985)
	46	Ctes. Ituzaingó, Garapé, Cerutti 68	
*Miltonia flavescens* (Lindl.) Lindl.	60	Mnes. Eldorado, Valle Hermoso, Honfi 1153	60 - Sinotô (1962, 1969), Charanasri *et al.* (1973), Félix and Guerra (2000)
*Oeceoclades maculata* (Lindl.) Lindl.	56	Mnes. San Ignacio, Dematteis 486	56 - Dematteis and Daviña (1999)
	56	Mnes. Capital, Posadas, Daviña 613	48, *c.*52, 54, 58 - Guerra (1986), Felix and Guerra (2000)
*Oncidium bifolium* Sims	108	Mnes. Guaraní, Honfi 1359	108 - Dematteis and Daviña (1999)
*O. divaricatum* Lindl.^3^	56	Mnes. Bernardo de Irigoyen, Pozo Azul, Honfi 1360	42 - Charanasri *et al.* (1973)
*O. edwallii* Cogn.^1^	42	Mnes. Montecarlo, Puerto Rico, Insaurralde w/n	-
*O. fimbriatum* Lindl.^1^	56	Mnes. Bernardo de Irigoyen, Pozo Azul, Honfi 1363	-
*O. longicornu* Mutel ^3^	42	Mnes. Iguazú, Puerto Iguazú, Daviña 615	56 - Dematteis and Daviña (1999)
*O. longipes* Lindl.	56	Mnes. Iguazú, Puerto Iguazú, Daviña 616	56 - Blumenschein (1960), Dematteis and Daviña (1999)
*O. pubes* Lindl.^1^	84	Mnes. Guaraní, Cabassi w/n	-
*O. riograndense* Cogn.^1^	56	Mnes. Bernardo de Irigoyen, Pozo Azul, Honfi 1192	-
*Pelexia bonariensis* (Lindl.) Schltr.	46	Mnes. Capital, Nemesio Parma, Cerutti 29	46 - Martínez (1985), Dematteis and Daviña (1999)
	46	Mnes. Candelaria, P. P. Cañadón de Profundidad, Hojsgaard 289	
*P. ekmanii* (Kraenzl) Schltr.^1^	46	Mnes. Capital, Garupá, Radins 15	-
*P. lindmanii* Kraenzl ^1.^	46	Mnes. San Pedro, P. P. Saltos del Moconá, Daviña 123	-
*Rodriguezia decora* (Lem.) Rchb. f.	42	Mnes. Bernardo de Irigoyen, Pozo Azul, Honfi 1362	Sinoto (1962)
*Sacoila lanceolata* (Aubl.) Garay	46	Mnes. Capital, Garupá, Radins 55	46 - Cocucci (1956), Martínez (1985), Felix and Guerra (2005)
*Sarcoglottis fasciculata* (Vell.) Schltr.^3^	46, 47, 49	Mnes. Capital, Nemesio Parma, Hojsgaard 291B	46 - Martínez (1985), Felix and Guerra (2005)
	46	Mnes. Capital, Garupá, Insaurralde w/n	
	46	Ctes. Ituzaingó, Garapé, Insaurralde w/n	
*S. grandiflora* (Hook.) Klotzsch	46	Mnes. Capital, Nemesio Parma, Cerutti 56	46 - Martínez (1985)
	46	Ctes. Ituzaingó, Garapé, Insaurralde w/n	
*S. ventricosa* (Vell.) Hoehne	46	Mnes. Montecarlo, Isla Caraguatay, Hojsgaard 255	46 - Martínez (1985)
*Skeptrostachys paraguayensis* (Rchb. f.) Garay ^2^	46	Mnes. Apóstoles, San José, Baumgratz 19	-
*Sophronitis cernua* Lindl.	40	Mnes. Capital, Posadas, Honfi 1361	40 - Blumenschein (1960)
*Stigmatosema polyaden* (Vell.) Garay ^2^	40	Mnes. Apóstoles, San José, Hadad 18	-
*Trichocentrum pumilum* (Lindl.) M.W.Chase & N.H.Williams	30	Mnes. Capital, Villa Lanús, Cerutti 73	30 - Dematteis (1997), Dematteis and Daviña (1999), Felix and Guerra (2000)
*Warrea warreana* (Lodd. ex Lindl.) C. Schweinf.^1^	48	Mnes. San Pedro, P. P. Saltos del Moconá, Insaurralde w/n	-
*Zygopetalum maxillare* Lodd.	48	Mnes. Capital, Posadas, Daviña 614	48 - Blumenschein (1960), Tanaka and Kamemoto (1984)
*Zygostates alleniana* Kraenzl ^2^	54	Mnes. Apostoles, Aº Chimiray, Hojsgaard 343	-

^1^First chromosome number for the species, ^2^First chromosome number for the genus, ^3^Taxa with chromosome numbers that differ from previously published reports. Species are alphabetically grouped. All voucher specimens are deposited at MNES (Universidad Nacional de Misiones Herbarium). Mnes: Misiones, Ctes: Corrientes.
